# Supplementary material for: 68Ga-PSMA-PET/CT-based radiosurgery and stereotactic body radiotherapy for oligometastatic prostate cancer
Source: PLoS One. 2020 Oct 21;15(10):e0240892. doi: 10.1371/journal.pone.0240892 (PMC7577453; doi:10.1371/journal.pone.0240892)
Supplement: S1 Table — (DOCX) [file pone.0240892.s001.docx]

| **S1 Table. Metastasis location** | | |
| --- | --- | --- |
| **Metastasis number** | **Organ** | **Location** |
| 1 | Bone | Ilium |
| 2 | Bone | Sacrum |
| 3 | Bone | Th6 |
| 4 | Lymph node | pelvic |
| 5 | Lymph node | extra-pelvic |
| 6 | Lymph node | extra-pelvic |
| 7 | Lymph node | pelvic |
| 8 | Lymph node | pelvic |
| 9 | Lymph node | pelvic |
| 10 | Lymph node | pelvic |
| 11 | Bone | Pubis |
| 12 | Lymph node | extra-pelvic |
| 13 | Bone | Th7 |
| 14 | Lymph node | pelvic |
| 15 | Lymph node | pelvic |
| 16 | Bone | Th2 |
| 17 | Lymph node | Ilium |
| 18 | Bone | Th10 |
| 19 | Bone | Ilium |
| 20 | Bone | Costa 9 |
| 21 | Bone | Pubis |
| 22 | Lymph node | pelvic |
| 23 | Lymph node | extra-pelvic |
| 24 | Lymph node | pelvic |
| 25 | Bone | L1 |
| 26 | Lymph node | extra-pelvic |
| 27 | Lymph node | extra-pelvic |
| 28 | Bone | Costa 8 |
| 29 | Bone | Th2 |
| 30 | Bone | Ilium |
| 31 | Lymph node | extra-pelvic |
| 32 | Visceral | Lung |
| 33 | Bone | Th7 |
| 34 | Bone | Th9 |
| 35 | Bone | Th10 |
| 36 | Bone | Th12 |
| 37 | Bone | Ilium |
| 38 | Bone | Pubis |
| 39 | Bone | pelvic |
| 40 | Bone | Th11 |
| 41 | Lymph node | pelvic |
| 42 | Lymph node | pelvic |
| 43 | Lymph node | pelvic |
| 44 | Lymph node | pelvic |
| 45 | Lymph node | pelvic |
| 46 | Lymph node | pelvic |
| 47 | Lymph node | pelvic |
| 48 | Bone | Ilium |
| 49 | Lymph node | pelvic |
| 50 | Bone | Th11 |
| 51 | Lymph node | extra-pelvic |
| 52 | Lymph node | extra-pelvic |
| 53 | Lymph node | extra-pelvic |
| 54 | Lymph node | extra-pelvic |
| 55 | Lymph node | extra-pelvic |
| 56 | Lymph node | extra-pelvic |
| 57 | Bone | Costa 6 |
| 58 | Bone | L3 |
| 59 | Bone | Costa 6 |
| 60 | Lymph node | pelvic |
| 61 | Bone | L5 |
| 62 | Lymph node | pelvic |
| 63 | Bone | Costa 4 |
| 64 | Bone | Ilium |
| 65 | Bone | Sacrum |
| 66 | Lymph node | pelvic |
| 67 | Bone | Scapula |
| 68 | Bone | Costa 9 |
| 69 | Bone | Ilium |
| 70 | Bone | Costa 9 |
| 71 | Lymph node | extra-pelvic |
| 72 | Lymph node | pelvic |
| 73 | Lymph node | pelvic |
| 74 | Lymph node | pelvic |
| 75 | Bone | Femur |
