# Supplementary material for: 68Ga-PSMA-PET/CT-based radiosurgery and stereotactic body radiotherapy for oligometastatic prostate cancer
Source: PLoS One. 2020 Oct 21;15(10):e0240892. doi: 10.1371/journal.pone.0240892 (PMC7577453; doi:10.1371/journal.pone.0240892)
Supplement: S2 Table — (DOCX) [file pone.0240892.s002.docx]

| **S2 Table Treatment characteristics** | | | | | |
| --- | --- | --- | --- | --- | --- |
| **Metastasis number** | **PTV (cc)** | **PTV-surrounding prescription dose (Gy)** | **Fractions** | **Min dose (Gy)** | **Max dose (Gy)** |
| 1 | 5.10 | 24.0 | 1 | 23.81 | 34.29 |
| 2 | 3.49 | 20.0 | 1 | 19.19 | 26.24 |
| 3 | 14.05 | 16.0 | 1 | 10.76 | 17.18 |
| 4 | 7.40 | 21.0 | 1 | 20.26 | 30.00 |
| 5 | 23.10 | 24.0 | 3 | 20.16 | 34.29 |
| 6 | 23.10 | 24.0 | 3 | 20.16 | 34.29 |
| 7 | 6.78 | 24.0 | 3 | 21.43 | 34.29 |
| 8 | 13.24 | 19.0 | 1 | 17.86 | 27.14 |
| 9 | 1.80 | 21.0 | 1 | 19.83 | 29.66 |
| 10 | 1.70 | 21.0 | 1 | 20.17 | 30.00 |
| 11 | 9.40 | 20.0 | 1 | 18.81 | 28.57 |
| 12 | 4.02 | 24.0 | 3 | 22.22 | 34.28 |
| 13 | 16.90 | 24.0 | 3 | 19.37 | 34.29 |
| 14 | 2.48 | 19.2 | 3 | 17.63 | 24.95 |
| 15 | 4.75 | 19.2 | 3 | 18.06 | 24.94 |
| 16 |  | 21.0 | 1 |  |  |
| 17 | 7.18 | 19.0 | 1 | 19.19 | 27.14 |
| 18 | 1.67 | 20.0 | 1 | 17.82 | 28.57 |
| 19 | 3.96 | 20.0 | 1 | 20.01 | 28.57 |
| 20 | 1.55 | 20.0 | 1 | 18.90 | 28.57 |
| 21 | 2.87 | 20.0 | 1 | 17.61 | 22.81 |
| 22 | 0.89 | 20.0 | 1 | 19.20 | 28.57 |
| 23 | 2.69 | 24.0 | 3 | 22.70 | 34.29 |
| 24 | 3.32 | 28.8 | 6 | 33.62 | 37.04 |
| 25 | 8.81 | 21.0 | 1 | 21.10 | 30.00 |
| 26 | 13.64 | 20.0 | 1 | 19.03 | 28.57 |
| 27 | 2.87 | 21.0 | 1 | 19.74 | 30.00 |
| 28 | 5.63 | 20.0 | 1 | 20.12 | 28.57 |
| 29 | 5.67 | 19.0 | 1 | 15.23 | 27.14 |
| 30 | 21.06 | 20.0 | 1 | 18.69 | 28.57 |
| 31 | 2.47 | 22.0 | 1 | 21.97 | 31.43 |
| 32 | 41.05 | 25.0 | 1 | 22.35 | 35.71 |
| 33 | 1.37 | 21.0 | 1 | 18.97 | 30.00 |
| 34 | 2.44 | 21.0 | 1 | 19.38 | 29.52 |
| 35 | 5.65 | 21.0 | 1 | 19.89 | 30.00 |
| 36 | 1.26 | 21.0 | 1 | 18.50 | 28.12 |
| 37 | 2.98 | 20.0 | 1 | 19.17 | 28.57 |
| 38 | 10.75 | 20.0 | 1 | 18.73 | 28.57 |
| 39 | 10.81 | 25.0 | 5 | 20.29 | 35.71 |
| 40 | 37.45 | 19.0 | 1 | 12.63 | 27.14 |
| 41 | 2.23 | 20.0 | 1 | 18.61 | 28.53 |
| 42 | 2.04 | 20.0 | 1 | 19.09 | 27.44 |
| 43 | 1.90 | 20.0 | 1 | 19.80 | 26.99 |
| 44 | 3.01 | 20.0 | 1 | 20.24 | 28.57 |
| 45 | 2.21 | 18.0 | 1 | 18.01 | 25.71 |
| 46 | 7.54 | 18.0 | 1 | 17.06 | 25.71 |
| 47 | 1.37 | 22.0 | 1 | 21.67 | 31.43 |
| 48 | 62.27 | 18.0 | 1 | 16.33 | 25.71 |
| 49 | 3.61 | 18.0 | 1 | 17.21 | 25.71 |
| 50 | 5.27 | 19.0 | 1 | 12.45 | 27.14 |
| 51 | 12.03 | 20.0 | 1 | 18.43 | 28.57 |
| 52 | 5.54 | 20.0 | 1 | 18.79 | 28.57 |
| 53 | 3.75 | 20.0 | 1 | 18.55 | 25.35 |
| 54 | 1.66 | 21.0 | 1 | 19.54 | 29.28 |
| 55 | 3.91 | 21.0 | 1 | 18.97 | 30.00 |
| 56 | 1.65 | 21.0 | 1 | 20.01 | 29.42 |
| 57 | 11.77 | 20.0 | 1 | 19.24 | 28.57 |
| 58 | 4.74 | 20.0 | 1 | 19.18 | 28.57 |
| 59 | 17.58 | 21.0 | 1 | 20.55 | 30.00 |
| 60 | 5.03 | 24.0 | 3 | 23.08 | 30.26 |
| 61 | 7.32 | 20.0 | 1 | 19.61 | 28.57 |
| 62 | 3.56 | 20.0 | 1 | 18.99 | 28.57 |
| 63 | 13.46 | 20.0 | 1 | 18.96 | 28.57 |
| 64 | 2.45 | 21.0 | 1 | 19.47 | 30.00 |
| 65 | 48.70 | 19.0 | 1 | 17.60 | 27.14 |
| 66 | 1.44 | 18.0 | 1 | 15.19 | 25.71 |
| 67 |  | 24.0 | 3 |  |  |
| 68 | 14.06 | 19.0 | 1 | 18.43 | 27.14 |
| 69 | 5.20 | 21.0 | 1 | 20.81 | 30.00 |
| 70 | 4.92 | 19.0 | 1 | 18.43 | 27.14 |
| 71 | 1.86 | 20.0 | 1 | 18.85 | 28.57 |
| 72 | 3.11 | 24.0 | 3 | 21.83 | 34.28 |
| 73 | 9.42 | 24.0 | 3 | 19.41 | 34.28 |
| 74 | 9.42 | 24.0 | 3 | 19.41 | 34.28 |
| 75 | 2.71 | 18.0 | 1 | 18.07 | 28.57 |
